# Effects of Chromium-Loaded Chitosan Nanoparticles on the Intestinal Electrophysiological Indices and Glucose Transporters in Broilers

**DOI:** 10.3390/ani9100819

**Published:** 2019-10-17

**Authors:** Sajid Khan Tahir, Muhammad Shahbaz Yousaf, Sohrab Ahmad, Muhammad Khurram Shahzad, Ather Farooq Khan, Mohsin Raza, Khalid Abdul Majeed, Abia Khalid, Hafsa Zaneb, Imtiaz Rabbani, Habib Rehman

**Affiliations:** 1 Department of Physiology, University of Veterinary and Animal Sciences, Lahore 54000, Pakistan; sajid.tahir@uvas.edu.pk (S.K.T.); sohrabdvm@gmail.com (S.A.); khurram142@gmail.com (M.K.S.); mohsin.raza@uvas.edu.pk (M.R.); khalid.majeed@uvas.edu.pk (K.A.M.); khalidabia29@gmail.com (A.K.); imtiaz.rabbani@uvas.edu.pk (I.R.); habibrehman@uvas.edu.pk (H.R.); 2 Interdisciplinary Research Centre in Biomedical Materials, COMSATS University Islamabad, Lahore Campus, Lahore 45550, Pakistan; atherfarooq@ciitlahore.edu.pk; 3 Department of Anatomy and Histology, University of Veterinary and Animal Sciences, Lahore 54000, Pakistan; hafsa.zaneb@uvas.edu.pk

**Keywords:** nanoparticles, chromium, supplementation, electrophysiology, Ussing chamber, poultry

## Abstract

**Simple Summary:**

Chromium is an important trace element responsible for the metabolism of glucose by enhancing insulin activity. This study was planned to evaluate the effects of chromium-loaded chitosan nanoparticles on the transport of glucose or amino acid across jejunum, gene expression of glucose transporters, and glycogen contents of liver and muscle. The results revealed that an increase in the supplemented dose of chromium-loaded chitosan nanoparticles decreased liver glycogen content and glucose transport across jejunum, while the muscle glycogen, gene expression of glucose transporters, and amino acid transport remained unaffected.

**Abstract:**

The present study aimed to evaluate the effect of chromium-loaded chitosan nanoparticles (Cr-CNPs) on the electrophysiological indices, gene expression of glucose transporters, and tissue glycogen in broilers. A total of 200 one-day-old broilers were randomly divided into five groups, with each having five replicates (n = 8). Group A was fed a corn–soybean meal diet, while the diets of groups B, C, D, and E were supplemented with 200, 400, 800, and 1200 µg/kg of Cr as Cr-CNPs, respectively. On day 35, the jejunum was collected for electrophysiological study, gene expression of glucose transporters, and tissues glycogen determination. The basal short-circuit current and tissue conductance before the addition of glucose was the same in all groups. Following the addition of glucose, the change in short-circuit current decreased (*p* < 0.05) in the jejunal tissues of birds supplemented with 400 and 1200 µg Cr-CNPs compared with the control group. Gene expression of SGLT-1 and GLUT-2 remained unaffected with supplementation. The serum glucose and liver glycogen concentration decreased (*p* < 0.05) linearly with supplementation, while no effect was observed on muscle glycogen. In conclusion, Cr-CNPs supplementation decreases the glucose absorption and liver glycogen content, without affecting the gene expression of glucose transporters.

## 1. Introduction

Glucose metabolism of avian species differs from that of mammals; i.e., the birds have higher blood glucose concentration and lower insulin levels [1]. Furthermore, avian species are considered less sensitive to insulin than mammals [2]. Chromium (Cr), a trace element, is known to increase insulin sensitivity in mammals [3] whereas, in broilers, Cr supplementation has reduced the serum glucose concentration [4]. The Cr level in poultry feed is yet to be appropriately recommended [5]. It is believed that poultry diets containing Cr can meet the requirements of the birds reared under the standard management conditions specific to broilers. However, several studies reported a positive effect of Cr supplementation on the production performance and carcass traits of broilers [6,7,8].

Intestinal absorption of carbohydrates occurs via glucose transporters [9]. In birds, most of the glucose or amino acid transport occurs in the jejunum [10]. The sodium-dependent glucose co-transporter transports glucose, along with sodium, from the intestinal lumen, while the sodium-independent glucose transporter is responsible for the transport of glucose along the basolateral side [11]. The transport of Glucose and amino acid through the intestine can be evaluated by studying the electrical variables with the help of an Ussing chamber. The effect of chromium-loaded chitosan nanoparticles (Cr-CNPs) on the electrophysiological indices and gene expression of glucose transporters is yet to be reported in poultry. Limited studies are available regarding the effect of organic chromium picolinate and chromium histidinate on the glucose transporters in layers exposed to heat stress [9]. The current research is aimed to explore the effect of Cr-CNPs supplementation on the electrophysiological parameters, gene expression of glucose transporters, and tissue glycogen content in broilers reared under standard management conditions.

## 2. Materials and Methods

All the experimental procedures used were approved by the Ethical Review Committee of the University of Veterinary and Animal Sciences, Lahore, Pakistan, vide letter No. DR/498.

### 2.1. Preparation of Chromium-Loaded Chitosan Nanoparticles

The Cr-CNPs were prepared and characterized at the Interdisciplinary Research Center in Biomedical Research, COMSATS University Islamabad (Lahore Campus), Pakistan, according to the method described by Wang et al. [12]. Briefly, 1% (*w/v*) solution of chitosan was prepared by dissolving chitosan into a 0.5% acetic acid solution with the pH adjusted to 3.5. The chitosan solution was then stirred for one hour. During stirring, 200 mg/L of chromium chloride solution was added to the chitosan solution to get a suspension of chitosan and chromium chloride. The pH of the suspension was adjusted to 6.5, and stirring was continued for five hours. Subsequently, the precipitate was centrifuged at 12,000 g for 15 min at room temperature and washed with distilled water to get Cr-CNPs.

### 2.2. Experimental Animals, Grouping, Diet, and Management

Two hundred male broiler chicks (Hubbard), were randomly divided into five groups, with each having five replicates (n = 8). Birds in group A (control) were given the non-supplemented basal diet, as shown in Table 1 [13], while the birds in the groups B, C, D, and E were fed the same diet but supplemented with graded levels of Cr-CNPs i.e., 200, 400, 800, and 1200 µg/kg of Cr as Cr-CNPs, respectively, for 35 days. The feed and water were provided ad libitum. Temperature and relative humidity on day 1 was kept at 35 ± 1.1 °C and 65 ± 5%, respectively. The temperature was decreased by 3 °C per week until it reached 26 °C on day 21.

### 2.3. Sample Collection and Processing of Tissue

On day 35, eight birds per group were randomly selected and their jejunal segments were prepared as described earlier by Rehman et al. [14]. Briefly, after exsanguination, a segment of jejunum was removed, washed thoroughly with an ice-cold Ringer’s buffer solution, and transferred to the laboratory in buffer. The serosal layer was stripped off the jejunum. The jejunum was subsequently opened longitudinally along the mesenteric border, rinsed with Ringer’s solution to remove the luminal contents, and then gassed with carbogen (95% O_2_ and 5% CO_2_) till mounting on the Ussing chamber.

Blood samples were taken to determine the glucose level. One hundred milligrams of tissue from each liver and pectoral muscle was collected for quantification of glycogen contents. For mRNA quantification of glucose transporters, the collected jejunal segments were washed with ice-cold normal saline. All the samples were stored at −80 °C for further analyses.

### 2.4. Measurement of Electrophysiological Indices

The jejunal mucosa (with stripped-off serosal layer) was cut into four pieces of 1 cm^2^ and mounted in between two compartments of the Ussing chamber. The exposed area of the chamber was 0.95 cm^2^ [15]. Damage to the edge of the tissue was minimized by silicon rubber rings on both sides of the tissue. Buffer solution (16 mL) was added to the chambers on each side. The buffer solution contained (in mM) 1.2 CaCl_2_, 115 NaCl, 25 NaHCO_3_, 20 Mannitol, 5 KCl, 2.4 Na_2_HPO_4_, 1.2 MgCl_2_, and 0.4 NaH_2_PO_4_, with the pH adjusted to 7.4. The buffer solution was continuously aerated with carbogen (95% O_2_ and 5% CO_2_) and maintained at 37 °C. Buffer osmolarity was measured (Osmomat 030, Gonotec GmbH, Berlin, Germany) and adjusted to 300 mOsmol/L, using mannitol. Tissues were allowed to equilibrate for 20 min under open circuit and then short-circuited by clamping the voltage at 0 mV for 5 min. Following equilibrium, 10.0 mM glucose or L-glutamine was added to the mucosal side, and the peak electrical response was measured. Electrical measurements like short-circuit current (Isc) and transmural tissue conductance (Gt) were observed with an automatic computer-controlled voltage-clamp device (Mussler, Aachen, Germany) to assess the electrogenic transport of glucose or L-glutamine linked to sodium across the jejunal mucosa.

### 2.5. Extraction of RNA and Quantification of Glucose Transporters

The mRNA expression of glucose transporters (sodium-dependent glucose transporter-SGLT-1 and sodium-independent glucose transporter-GLUT-2) were determined by real-time PCR (Router gene 5 plex real-time PCR, Qiagen, Hilden, Germany). The oligonucleotide primers sequence for SGLT-1 [16], GLUT-2 [17], and ß-actin [17] (housekeeping gene) used for PCR amplification are shown in Table 2. The RNA extraction of the jejunal mucosa was done by using Trizol (Invitrogen, Karlsruhe, Germany), according to the manufacturer’s instructions. The total RNA was quantified by using a Nanodrop spectrophotometer (Thermo Fisher Scientific, Wilmington, DE, USA). A total of 5 µg RNA was reverse transcribed to complementary DNA (cDNA), using First-Strand cDNA synthesis kit (Thermo Scientific™, Waltham, MA, USA). Real-time PCR was performed using the SYBER green maxima PCR kit (Thermo Scientific™, Waltham, MA, USA), as per manufacturer’s instructions. The following PCR program was set on the machine to amplify the target mRNA in tissue extracts: 95 °C for 3 min, followed by 40 cycles of 95 °C for the 30 s, 60 °C for 30 s, and 72 °C for 30 s. To determine the melting points of the amplified cDNA and to confirm the production of a single product, a dissociation curve was generated after 40 cycles. Relative mRNA expression levels of SGLT-1 and GLUT-2 were determined using the 2^−ΔΔCT^ method [18].

### 2.6. Quantification of Tissues Glycogen and Serm Glucose

Liver and muscle glycogen contents were quantified by using iodine assay described by Bennet et al. [19] and Dreiling et al. [20], with some modifications. Briefly, 100 mg liver and muscle samples were homogenized using chilled perchloric acid to solubilize the glycogen. The homogenate was then centrifuged at 2500 rpm for 10 min at 4 °C. The supernatant was collected, and the pellet was re-homogenized with chilled perchloric acid. After another round of centrifugation, the supernatant thus collected was added to the previously collected supernatant and subjected to iodine assay. The absorbance of samples or glycogen standards was measured at 460 nm, using an EPOCH^TM^ microplate spectrophotometer (Biotek Instruments Inc., Winooski, VT, USA). The serum glucose concentration was estimated by the commercially available kit (DiaSys, Germany Germany), according to manufacturer’s instruction using the same EPOCH^TM^ microplate spectrophotometer.

### 2.7. Statistical Analysis

Data were statistically analyzed using Statistical Package for Social Sciences (SPSS for windows version 20.0, IBM). Data were presented as means ±SEM and were analyzed using one-way analysis of variance (ANOVA). For group differences, Tukey’s post hoc test was used. Polynomial contrasts were used to determine the linear, quadratic, and cubic effects of Cr-CNPs supplementation at *p* < 0.05.

## 3. Results

**Figure 1:**
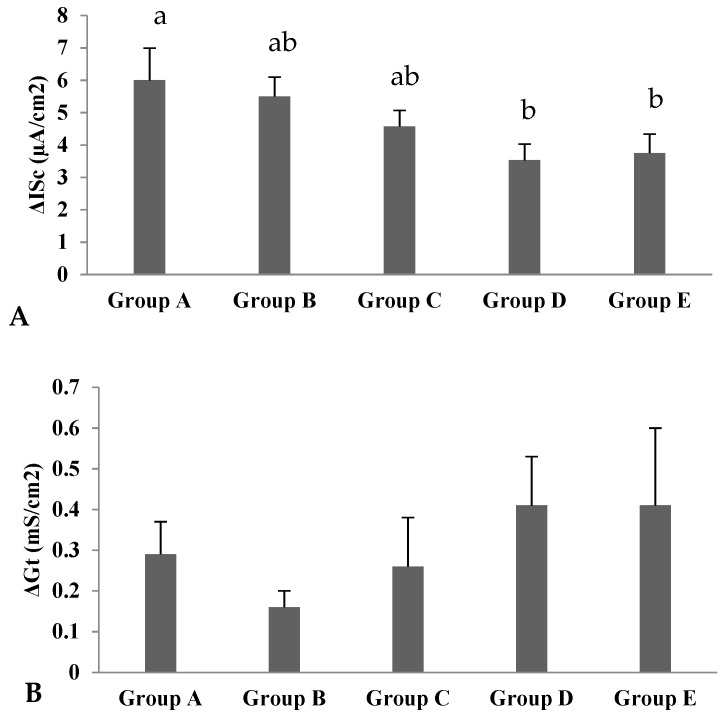
Effect of Cr-CNPs supplementation on (**A**) change in short-circuit current (ΔISc) and (**B**) change in tissue conductance (ΔGt) after addition of glucose to the jejunum in broilers. Labeled bars without a common letter differ significantly, *p* < 0.05. Data are presented as Mean ± SEM. Group A = control—without Cr-CNPs supplementation. Group B = offered 200 µg Cr-CNPs/kg of feed. Group C = offered 400 µg Cr-CNPs/kg of feed. Group D = offered 800 µg Cr-CNPs/kg of feed. Group E = offered 1200 µg Cr-CNPs/kg of feed.

The basal short-circuit current (Isc) and transmural tissue conductance (Gt) before the addition of glucose did not vary between the supplemented groups and the control group (Table 3). After the addition of glucose to the mucosal side, the change in short-circuit current decreased linearly (*p* < 0.05) in the groups C and E compared with the control group (Figure 1). However, no effect on the change in transmural tissue conductance was observed with Cr-CNPs supplementation after addition of glucose (Figure 1).

**Figure 2:**
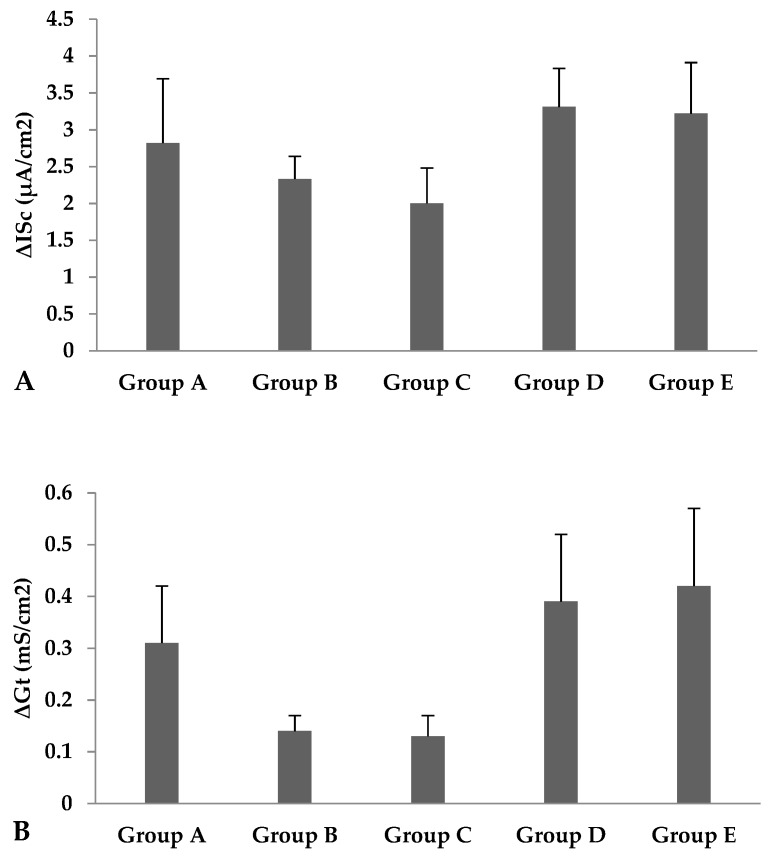
Effect of Cr-CNPs supplementation on (**A**) change in short circuit current (ΔISc) and (**B**) change in tissue conductance (ΔGt) after addition of L-glutamine to the jejunum in broilers. Mean values with different small letters on the same bar differ significantly at *p* < 0.05. Data are presented as Mean ± SEM. Group A = control—without Cr-CNPs supplementation. Group B = offered 200 µg Cr-CNPs/kg of feed. Group C = offered 400 µg Cr-CNPs/kg of feed. Group D = offered 800 µg Cr-CNPs/kg of feed. Group E = offered 1200 µg Cr-CNPs/kg of feed.

Prior to the addition of glutamine addition, the basal Isc and Gt were similar in all supplemented groups compared to the control group (Table 4). After the addition of glutamine to the mucosal side, no effect of Cr-CNPs supplementation was observed on the change in short-circuit current and change in transmural tissue conductance, as shown in Figure 2.

**Figure 3:**
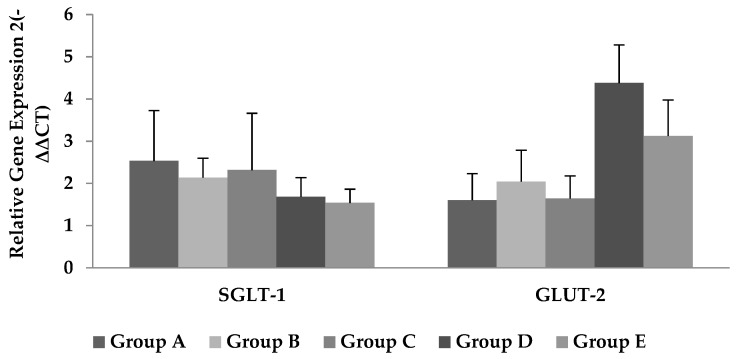
Effect of Cr-CNPs supplementation on gene expression of glucose transporters, i.e., (left side) SGLT-1, and (right side) GLUT-2 in broilers. Data are presented as mean ± SEM. Group A = control—without Cr-CNPs supplementation. Group B = offered 200 µg Cr-CNPs/kg of feed. Group C = offered 400 µg Cr-CNPs/kg of feed. Group D = offered 800 µg Cr-CNPs/kg of feed. Group E = offered 1200 µg Cr-CNPs/kg of feed.

The mRNA expression of glucose transporters, i.e., SGLT-1 and GLUT-2, remained unaffected by the Cr-CNPs supplementation compared to the control group, as shown in Figure 3.

The liver glycogen concentration in groups D and E decreased linearly (*p* < 0.05) with the Cr-CNPs supplementation compared with the control group. No significant effect of Cr-CNPs supplementation was found on muscle glycogen concentration (Table 5). The serum glucose level decreased linearly (*p* < 0.05) with the Cr-CNPs supplementation (Table 5).

## 4. Discussion

Chromium (Cr) is a biologically active trace element that plays a key role in metabolic activities in the body. Upon absorption, Cr is found in the blood either in free form or in bound-to-globulin proteins, transferrin, or complexes like glucose tolerance factor [21]. The bioavailability of organic chromium is higher than the inorganic form due to its increased absorption rate [22]. The inorganic form irreversibly binds with the undigested content in the intestine, and, hence, its absorption and bioavailability are limited [23]. The absorption of Cr can be enhanced by chelation, as it prevents the precipitation of chromium at alkaline pH in the poultry intestine [24].

The intestine of birds is highly absorptive for water and electrolytes. The electrical current across the epithelium is due to the net movement of ions. The transport of glucose and amino acids across the membrane occurs by either the paracellular or transcellular pathway [14]. Paracellular transport occurs without the expense of energy, while transcellular transport utilizes energy to transport glucose via sodium-dependent glucose transport (SGLT-1) and amino acids by carrier proteins in the luminal and basolateral membranes [25]. Transcellular transport of glucose and amino acids in poultry occurs in the small intestine and colon [26,27]. Most of the sodium-dependent uptake of glucose and amino acids via carrier proteins takes place in the jejunum [10]. The addition of glucose and amino acids to the luminal side of the intestine potentiates carrier-mediated transport, along with the enhanced uptake of luminal sodium. In response, the intestinal membrane depolarizes, and increased cytoplasmic sodium stimulates the sodium–potassium ATPase pump in the basolateral membrane, which, in turn, increases the net movement of sodium from the mucosal to the serosal side. These events bring changes in the electrical variable of the intestine and increase the short-circuit current [14,28,29]. In the present study, the basal short-circuit current and tissue conductance remained unaffected with Cr-CNPs supplementation before the addition of glucose or glutamine, which indicates good preservation and preparation of the tissues [30]. After the addition of glucose to the mucosal side, the change in short-circuit current (ΔISc) decreased linearly in jejunal tissues of Cr-CNPs-supplemented birds, but no effects were observed in ΔISc upon the addition of glutamine to the mucosal side. The decline in ΔISc after glucose addition reflects a decrease in sodium transport across the intestinal membrane. The change in tissue conductance did not vary in tissues of Cr-CNPs-supplemented birds after the addition of glucose or glutamine. To the best of our knowledge, no data are available regarding the effects of the Cr-CNPs on the electrophysiological indices in poultry. Gammelgaard et al. [31] conducted in vitro permeation studies with an Ussing chamber by using pig intestine to compare the absorption of organic and inorganic chromium. They found no response due to adsorption of chromium to the chambers.

The uptake of carbohydrates at the level of the intestine is facilitated by glucose transporters. The GLUT-2 is responsible for the exit of monosaccharides from the enterocytes by facilitated diffusion, while the SGLT-1 mediates the uptake of monosaccharides [32]. The SGLT-1 is expressed in the intestine and kidney [33]. It was reported that glucose absorption was decreased in SGLT-1-deficient mice, which depicts the role of SGLT-1 in maintaining the sodium–glucose homeostasis [15]. In our study, no effect was observed on the expression of GLUT-2 and SGLT-1 with the Cr-CNPs supplementation. Contrary to our study, Orhan et al. [9] reported an increase in the expression of SGLT-1 and GLUT-2 with chromium picolinate and chromium histidinate in layers subjected to heat-stress conditions. The possible reasons could be difference in chromium sources or environmental conditions. In our study, the ΔISc on mucosal addition of glucose decreased linearly with Cr-CNPs supplementation, but gene expression of SGLT-1 remained unaffected. The expression of GLUT-2 is, however, upregulated (*p* > 0.05) with the increase in Cr-CNPs concentration, which might have facilitated transportation of other monosaccharides, including fructose or galactose. However, translation of mRNA to GLUT-2 protein is still debatable and calls for further investigations into the role of Cr-CNPs. It may be due to the lack of consistency between mRNA and protein concentration data [34].

Chromium is a cofactor of glucose tolerance factor and enhances insulin function to increase the cellular uptake of glucose [35]. In our study, the liver glycogen concentration decreased linearly with Cr-CNPs supplementation, but no effect of Cr-CNPs supplementation was found on muscle glycogen concentration. Brooks et al. [1] reported no effects on muscle glycogen or liver glycogen in broilers supplemented with 200, 400, or 800 μg/kg of Cr as chromium propionate. Also, Cr supplementation at 1 mg/kg diet to a low-Cr diet increased liver glycogen synthetase activity but did not affect liver glycogen concentrations in rats [36]. Chromium supplementation also did not affect muscle glycogen in broilers [37], rats [36], humans [38], and sheep [39]. In birds, the liver is the major site of glycogen storage, and liver glycogen is a readily available source of glucose for homeostasis [19]. In our study, the linear decrease in liver glycogen may be the result of glycogenolysis in order to maintain homeostasis that was affected by the linear decrease in glucose absorption from the intestine following Cr-CNPs supplementation.

## 5. Conclusions

In conclusion, Cr-CNPs supplementation decreases the absorption of glucose across the jejunal mucosa, with a concomitant decrease in liver glycogen concentration. However, it does not affect the expression of glucose transporters. Further insights are required to explore the effect of Cr-CNPs on the pathways involved in glucose metabolism and transportation.

## Figures and Tables

**Table 1:** Composition of the diet (g/kg).

Ingredients	Percentage
Corn	58.50
Soybean meal 44%	25.00
Sunflower meal	3.50
Canola meal	8.00
Vegetable oil	1.50
Dicalcium phosphate	0.90
Limestone	1.51
Common salt	0.50
DL-Methionine	0.21
L-Lysine HCl	0.12
Vitamin premix ^1^	0.13
Micro mineral premix ^2^	0.13
Total	100.00
**Nutrient contents**	
Crude protein	20.72
Metabolizable energy (MJ)	12.2
Calcium	0.91
Phosphorus	0.61
* Available P	0.33

^1^ Provided vitamins per kg of the feed: vitamin A (retinol), 11000 IU; vitamin B-12 (cyanocobalamin), 0.0132 mg; vitamin D_3_ (cholecalciferol), 2200 IU; vitamin E (alpha-tocopherol), 22 IU; choline chloride, 440 mg; riboflavin, 8.8 mg; pantothenic acid, 22 mg; ethoxyquin, 250 mg; menadione, 2.2 mg; pyridoxine, 4.4 mg; folic acid, 1.1 mg; biotin, 0.22; thiamin, 4.4 mg. ^2^ Supplied minerals per kg of the feed: Cu (CuSO_4_), 20 mg; Zn (ZnO), 200 mg; Mn (MnSO_4_), 240 mg; Fe (FeSO_4_), 120 mg; I (KI), 0.92 mg; Ca, 150–180 mg. Measured Chromium 4.05 mg/kg. * Calculated according to NRC 1994.

**Table 2:** Primer sequences used during real-time PCR.

Name of Gene	Forward Primer (5′–3′)	Reverse Primer (5′–3′)	Annealing Temperature (°C)
SGLT-1	GTCCTGGCAGTGGGAGTATG	AAGAGTGAAGCACCGATCGG	61
GLUT-2	CACACTATGGGCGCATGCT	ATTGTCCCTGGAGGTGTTGGTG	60
β-Actin	ATGAAGCCCAGAGCAAAAGA	GGGGTGTTGAAGGTCTCAAA	60

SGLT-1 (Na^+^-dependent glucose and galactose transporter); GLUT-2 (Na^+^-independent glucose, galactose and fructose transporter).

**Table 3:** Effect of Cr-CNPs supplementation on initial short-circuit current and tissue conductance before the addition of glucose.

Variables	Group A	Group B	Group C	Group D	Group E	SEM	*p*-Value	Linear	Quadratic	Cubic
ISci (µA/cm^2^)	7.71	5.57	6.81	4.93	4.08	0.50	0.158	0.095	0.746	0.077
Gti (mS/cm^2^)	4.24	2.90	2.72	4.53	3.51	0.28	0.135	0.935	0.260	0.039

Data are presented as Mean ± SEM. Group A = control—without Cr-CNPs supplementation. Group B = offered 200 µg Cr-CNPs/kg of feed. Group C = offered 400 µg Cr-CNPs/kg of feed. Group D = offered 800 µg Cr-CNPs/kg of feed. Group E = offered 1200 µg Cr-CNPs/kg of feed. ISci = initial short circuit current. Gti = initial tissue conductance.

**Table 4:** Effect of Cr-CNPs supplementation on initial short-circuit current and tissue conductance before the addition of L-glutamine.

Variables	Group A	Group B	Group C	Group D	Group E	SEM	*p*-Value	Linear	Quadratic	Cubic
ISci (µA/cm^2^)	9.55	6.67	6.67	9.56	8.67	0.62	0.353	0.812	0.221	0.129
Gti (mS/cm^2^)	4.50	3.09	2.78	4.45	4.45	0.29	0.057	0.415	0.080	0.059

Data are presented as Mean ± SEM. Group A = control—without Cr-CNPs supplementation. Group B = offered 200 µg Cr-CNPs/kg of feed. Group C = offered 400 µg Cr-CNPs/kg of feed. Group D = offered 800 µg Cr-CNPs/kg of feed. Group E = offered 1200 µg Cr-CNPs/kg of feed. ISci = initial short circuit current. Gti = initial tissue conductance.

**Table 5:** Effect of Cr-CNPs supplementation on liver and muscle glycogen concentration (mg/100g) and blood glucose concentration (mg/dL) in broilers.

Variables	Group A	Group B	Group C	Group D	Group E	SEM	*p*-Value	Linear	Quadratic	Cubic
Liver	13.38 ^a^	13.89 ^a^	8.71 ^ab^	7.36 ^b^	6.71 ^b^	0.93	0.014	0.002	0.826	0.230
Muscle	3.37	3.75	3.11	3.24	3.05	0.17	0.756	0.382	0.807	0.592
Blood Glucose	252 ^a^	267 ^a^	229 ^a^	218 ^ab^	164 ^b^	14.5	<0.001	<0.001	0.046	0.796

Data are presented as mean ± SEM. ^a–b^ Within the same row, different superscript indicates significantly different means at *p* < 0.05. Group A = control—without Cr-CNPs supplementation. Group B = offered 200 µg Cr-CNPs/kg of feed. Group C = offered 400 µg Cr-CNPs/kg of feed. Group D = offered 800 µg Cr-CNPs/kg of feed. Group E = offered 1200 µg Cr-CNPs/kg of feed.
